# Blocking Tactile Input to One Finger Using Anaesthetic Enhances Touch Perception and Learning in Other Fingers

**DOI:** 10.1037/xge0000514

**Published:** 2019-04

**Authors:** Harriet Dempsey-Jones, Andreas C. Themistocleous, Davide Carone, Tammy W. C. Ng, Vanessa Harrar, Tamar R. Makin

**Affiliations:** 1 Institute of Cognitive Neuroscience, University College London, and the Oxford Centre for Functional MRI of the Brain (FMRIB), Nuffield Department of Clinical Neuroscience, University of Oxford; 2 Nuffield Department of Clinical Neurosciences, University of Oxford, and Brain Function Research Group, School of Physiology, Faculty of Health Sciences, University of the Witwatersrand; 3 Acute Vascular Imaging Centre, Radcliffe Department of Medicine, University of Oxford, and Laboratory of Experimental Stroke Research, Department of Surgery and Translational Medicine, University of Milano Bicocca, Milan Center of Neuroscience; 4 Department of Anaesthesia, University College Hospital; 5 Visual Psychophysics and Perception Laboratory, School of Optometry, and NSERC-Essilor Industrial Research Chair, University of Montreal; 6 Institute of Cognitive Neuroscience, University College London, and the Oxford Centre for Functional MRI of the Brain (FMRIB), Nuffield Department of Clinical Neuroscience, University of Oxford

**Keywords:** anesthetic, deafferentation, plasticity, psychophysics, somatosensory

## Abstract

Brain plasticity is a key mechanism for learning and recovery. A striking example of plasticity in the adult brain occurs following input loss, for example, following amputation, whereby the deprived zone is “invaded” by new representations. Although it has long been assumed that such reorganization leads to functional benefits for the invading representation, the behavioral evidence is controversial. Here, we investigate whether a temporary period of somatosensory input loss to one finger, induced by anesthetic block, is sufficient to cause improvements in touch perception (“direct” effects of deafferentation). Further, we determine whether this deprivation can improve touch perception by enhancing sensory learning processes, for example, by training (“interactive” effects). Importantly, we explore whether direct and interactive effects of deprivation are dissociable by directly comparing their effects on touch perception. Using psychophysical thresholds, we found brief deprivation alone caused improvements in tactile perception of a finger adjacent to the blocked finger but not to non-neighboring fingers. Two additional groups underwent minimal tactile training to one finger either during anesthetic block of the neighboring finger or a sham block with saline. Deprivation significantly enhanced the effects of tactile perceptual training, causing greater learning transfer compared with sham block. That is, following deafferentation and training, learning gains were seen in fingers normally outside the boundaries of topographic transfer of tactile perceptual learning. Our results demonstrate that sensory deprivation can improve perceptual abilities, both directly and interactively, when combined with sensory learning. This dissociation provides novel opportunities for future clinical interventions to improve sensation.

Long-term sensory input loss (hereafter, “deafferentation”) is known to trigger brain reorganization. For example, individuals born without eyesight show occipital lobe activity for various nonvisual tasks (e.g., auditory and tactile tasks; Sathian, 2005; Sathian & Stilla, 2010). Similarly, adults with upper-limb amputation recruit the missing-hand area during movement of their intact hand (Makin et al., 2013; Philip & Frey, 2014). It is commonly assumed that the invading representations can directly benefit from the freed-up cortical territory, leading to functional advantages for perception and action (Bottari, Nava, Ley, & Pavani, 2010; Merabet & Pascual-Leone, 2010; Nava & Röder, 2011).

However, previous evidence supporting such functional advantages has been challenged (see review in Makin & Bensmaia, 2017). For instance, enhanced tactile perception in the blind may be related to greater experience with, or dependence on, touch (Grant, Thiagarajah, & Sathian, 2000; Heller, 1989) as opposed to the recruitment of visual areas by tactile processes (see Kupers & Ptito, 2014, for review). Vega-Bermudez and Johnson (2002) convincingly demonstrated that amputation of a finger does not confer tactile gains on the adjacent fingers (see also Oelschläger, Pfannmöller, Langner, & Lotze, 2014). Moreover, others have suggested that deprivation-related reorganization in adults has maladaptive sensory consequences (Flor et al., 1995; Haak, Morland, & Engel, 2015). Thus, the functional consequences of long term deprivation-related reorganization remain unclear.

Studies investigating improvement of sensorimotor abilities by temporary, experimentally induced sensory deafferentation have been similarly inconclusive. Although some studies showed tactile improvements across measures (Weiss et al., 2011; Werhahn, Mortensen, Van Boven, Zeuner, & Cohen, 2002), most studies have shown limited improvement in measures of touch perception (Björkman, Rosén, & Lundborg, 2004; Björkman, Weibull, Rosén, Svensson, & Lundborg, 2009; Weiss, Miltner, Liepert, Meissner, & Taub, 2004) or motor performance (Floel et al., 2004; both sensory and motor, see Rosen, Björkman, & Lundborg, 2006), or no change in any touch measure tested (in a healthy control group, see Sens et al., 2013; see also Björkman et al., 2004, for improvement in touch but not motor performance). A key consideration when interpreting such reports is that perceptual changes may not be caused by the deafferentation per se but instead by exposure to testing protocols (unintentional “training”) or by altered behavior also triggered by the deafferentation (see Discussion). Thus, it is difficult to dissociate the contributions of deafferentation alone to perceptual changes from the currently existing body of research.

Extending this concept, some groups have investigated whether deprivation-related plasticity can be harnessed to explicitly boost sensory *training* effects. For example, can deprivation enhance perceptual learning—the inherent ability of sensory systems to improve in perception following repeated exposure to stimuli or direct training (Gibson, 1969)—or motor learning—improvements in motor performance by practice or training (Schmidt & Lee, 2011)? Here again, results have been mixed, with reports that temporary deafferentation either improves motor learning (Muellbacher et al., 2002; Ziemann, Muellbacher, Hallett, & Cohen, 2001) or improves tactile learning (Voller et al., 2006) but not motor learning (through rehabilitation; Rosen et al., 2006; Weiss et al., 2011). In vision, 3 days of monocular deprivation has been reported to enhance contrast sensitivity training in the nondeprived eye (Shibata, Kawato, Watanabe, & Sasaki, 2012). However, the question still remains whether these gains represent “direct” effects of deafferentation (e.g., resulting from increased cortical representation; Merzenich, Kaas, Wall, Nelson, et al., 1983, 1984) or are induced via “interactive” effects of deafferentation (e.g., by facilitating ongoing processes of sensory plasticity such as perceptual learning).

Here, we wished to determine whether sensory deafferentation to one finger can enhance tactile perception directly as well as interactively—by improving the efficacy of a tactile training protocol. Further, we wished to investigate whether these direct and interactive effects of deafferentation are dissociable, delineating their separate contributions to perceptual gains. Because previous studies typically fail to include a deafferentation-only control condition, to our knowledge, such a dissociation has not yet been successfully demonstrated.

We compared changes in tactile perception over time in three groups: one group experienced two (1-hr) pharmacological nerve blocks to the right index finger, carried out on subsequent days—the “Block-only” group. In two additional groups, tactile training was performed on the finger next to the blocked (or sham blocked) finger—these were the Block + Train and Sham + Train groups, respectively. Tactile perception was tested at multiple time points before and after the blocks to examine the time course of perceptual changes caused by deafferentation and/or training.

We predicted that the block would cause enhancements of tactile perception largely (or completely) restricted to the finger adjacent to the deafferented finger. This could be achieved through the redistribution of neuronal resources that have been freed up as a result of deafferentation (e.g., see Faggin, Nguyen, & Nicolelis, 1997, and Discussion). Critically, we predicted these “direct” effects of deafferentation in the Block-only group would be largely finger specific, that is, improvements of touch perception would be largely restricted to the deafferentation-adjacent finger. This follows from studies showing the greatest physiological deafferentation changes for deafferentation-adjacent locations (e.g., recruitment of cortical territory proximal to the deafferented zone; Merzenich, Kaas, Wall, Nelson, et al., 1983; Merzenich et al., 1984). This prediction is visualised in Figure 1A.[Fig-anchor fig1]

Second, we predicted that training coupled with a sensory block (Block + Train group) would result in greater *transfer* of learning from the trained finger to the other fingers compared with sham block and training (Sham + Train group; see prediction in Figure 1, Panel B vs. C). A strong body of literature indicates that tactile learning transfers in a defined and highly consistent pattern, from the trained finger to the adjacent and homologous fingers only (in humans, see Dempsey-Jones et al., 2016, Harrar, Spence, & Makin, 2014, and Harris, Harris, & Diamond, 2001; in rodents, see Harris & Diamond, 2000; Harris et al., 1999; for review, see Tamè, Braun, Holmes, Farnè, & Pavani, 2016). This specific learning transfer pattern has been suggested to reflect topographic transfer in the somatosensory system, resulting from overlap in receptive fields (RFs; Harris et al., 2001). Critical to our prediction, learning has not been found to generalize to fingers other than the adjacent or homologous ones, presumably because of insufficient physiological overlap to permit transfer. Here, we wished to determine whether we could extend the boundary restricting the topographic spread of learning gains by deafferentation concurrent to training. Specifically, we investigated whether we could induce learning gains in the index and ring fingers of the untrained hand—fingers that do not normally showing benefits from learning transfer. Gains for these fingers would therefore be expected in the Block + Train group but not the Sham + Train group. Such a change could occur because the direct effects of deafferentation change neighborhood relationships between fingers in the somatosensory system (Faggin et al., 1997), thereby altering the transfer of learning (see Discussion).

Finally, and critically, we wished to show that extensive sensory gains in the Block + Train group were truly a result of the interaction of deafferentation and training and not simply a result of deafferentation alone. This would be reflected in divergent patterns of sensory gains in the Block + Train group compared with the Block-only group. We predicted learning in the deafferent-adjacent finger of both groups (resulting from the direct effects of deafferentation). However, we anticipated significantly more learning in the remaining five fingers for the Block + Train group compared with the Block-only group. Importantly, revealing a statistical divergence in the pattern of learning gains produced by these two groups would allow us to provide first evidence of a dissociation in direct and interactive effects of deafferentation.

## Method

### Participants

Forty-seven participants were recruited for the study. One participant dropped out of the study, six participants were excluded because of ineffective anesthesia, and four were removed from the analysis because of insufficient tactile perception (accuracy at chance on more than one finger at baseline testing). The remaining 36 participants were randomly allocated to one of three conditions based on order of sign-up (some final participants were directly assigned to groups to ensure age and gender matching across groups). There were three test groups of 12 participants each: block only (age, *M* = 26.25, standard error of the mean [*SEM*] = 1.45; seven females; no left-handers), Sham + Train (age, *M* = 27.17, *SEM* = 1.50; seven females; one left-hander), and Block + Train (age, *M* = 29.92, *SEM* = 2.59; five females; one left-hander).

All participants provided written informed consent prior to participation. Ethical approval was granted by the National Health Service Research Authority (reference code: 13/SC/0502). Participants were reimbursed for their time. Exclusion criteria included allergy to local anesthetic, medical or physical issues causing impaired perception to the fingertips, history of neurological or psychiatric illness, history of drug abuse, major illness within the last 3 months, pregnancy, and needle phobia.

### Experimental Timeline

**Figure 2 fig2:**
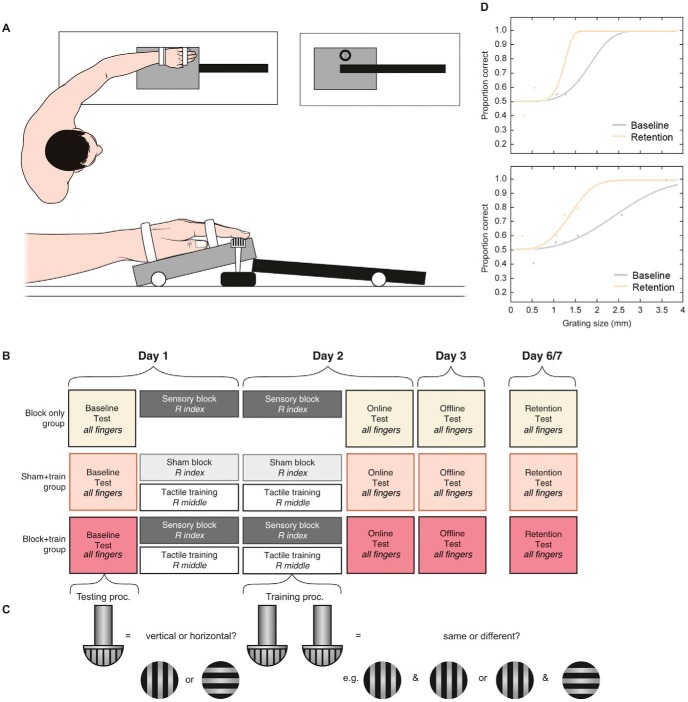
(A) Apparatus for presentation of experimental stimuli (tactile dome gratings)—views from the top and side. (B) Schedule of testing and training for the three groups. Please note that in the testing task, “all fingers” refers to all six fingers tested, that is, the index, middle, and ring fingers of the left and right hands. (C) Schematic description of the testing and training tasks, with grating orientations. (D) Example psychometric functions from two representative participants of the Block-only group (tactile “threshold” corresponds with the interpolation of 82% accuracy on the *y*-axis). The psychometric functions show threshold improvement for the (deafferentation-adjacent) right middle finger from the baseline to retention tests, that is, the direct effects of sensory block on the adjacent finger. Improvement in perception is reflected by a drop in grating orientation threshold (lower grating size values on the *x*-axis).

The study was conducted over a period of 6 to 7 days. The experimental time course is shown in Figure 2B. Day 1 involved a baseline test, followed by a real/sham block. Once an effective block was achieved (indicated by a sensitivity check; see section: Sensitivity Check), trained groups underwent the first session of minimal tactile training to the right middle finger (i.e., concurrent block and training). During this time, the Block-only group was allowed a supervised break. Day 2 involved a real/sham deafferentation and sensitivity check, followed by a second training session (or another supervised break for the Block-only group), and, finally, the online test. Day 3 involved the offline test alone. The final retention test was given 3 to 4 days subsequent to the offline test. Please note that the nomenclature of the testing sessions indicates the anticipated state of deafferentation effects. For instance, *online* infers there may have been some residual anesthetic effects at the time of testing (given the predicted duration of the block). *Offline* indicates anesthesia had ceased. *Retention* indicates anesthesia had ceased by an extended period.[Fig-anchor fig2]

### General Procedures

During training and testing, participants were blindfolded. They were instructed to prioritize accuracy over speed, and no time limit was imposed. Stimuli presentation was controlled by a computer running MATLAB (MathWorks, Inc., Boston, MA). During tasks, participants were asked to respond with a mouse using the index and middle fingers of the hand that was not being used for testing/training. Prior to the first testing session, participants in all groups were briefly familiarized with the testing protocol (approximately five presentations of the largest grating, in randomly alternating orientations with accompanying verbal labels). Trained groups received a similar familiarization prior to the first training (approximately five presentations of task-relevant stimuli conditions; see section: Testing Task). Note that stimuli and task details for testing and training were similar to those described in our previous studies (for more details, see Dempsey-Jones et al., 2016; Harrar et al., 2014).

### Deafferentation Interventions

All participants received the same deafferentation protocol. The intervention varied only in the substance injected, which depended on group assignment. With the participant’s right hand pronated, a trained physician inserted a 25-gauge sterile needle into the dorsolateral aspect of the base of the right index finger. One milliliter of solution was injected continuously as the needle was withdrawn. The same procedure was repeated on the other side of the base of the finger to achieve anesthesia of the entire finger (a “ring block”). The two blocked groups received an injection of lidocaine hydrochloride 1% and the sham group received normal saline 0.9%. The volume, type, and concentration of local anesthetic used provided a block duration of approximately 1 hr (lasting up to 3 hr). This blocking procedure prevents afferent sensory input from the finger, whereas motor function is largely preserved (because the tendons that allow finger movement reside in the hand/arm, outside the region of the nerve block). In the current study, we did not include a “sham only” condition (i.e., repeated testing alongside two sham blocks) to demonstrate the effect of testing alone on perceptual thresholds. This was because we have demonstrated in two previous studies (Dempsey-Jones et al., 2016; Harrar et al., 2014) that repeated testing does not cause selective changes in sensory thresholds of any one finger (gains are consistent across fingers over testing sessions; see Part 1 of the online supplemental materials for more information).

Two sessions of blocking (on subsequent days) were included in the protocol to maximize the effect of deafferentation. Participants in all groups were informed that they were receiving local anesthetic—but that the effects were variable and they therefore may not subjectively perceive a complete anesthetic effect (in debriefing, only one person in the sham group reported suspected administration of a sham block).

### Testing Task

The testing task assessed perception of grating orientation using a set of seven plastic dome gratings (JVP Domes; Stoelting, Wood Dale, IL). This test overcomes various pitfalls of other measures of tactile perception, such as two-point discrimination (see Tong, Mao, & Goldreich, 2013, and Van Boven & Johnson, 1994, for critique). The fingers tested were the index, middle, and ring fingers of the left and right hands. Because training was administered on the right middle finger, our selection of these six testing fingers allowed us to probe for gains in three fingers known to benefit from learning transfer (the adjacent index and adjacent ring on the trained hand, and the homologous middle finger of the untrained hand), as well as two fingers of no topographic relation to the trained finger—that consequently do not show learning transfer gains (the index and ring fingers of the trained hand; see the introduction).

The gratings varied in groove width, with isometric groove spacing (i.e., grooves and ridges were equal in diameter). The spacings were 0.25, 0.5, 1.0, 1.2, 1.5, 2.5, and 3.5 mm (with the smallest spacing being the hardest to feel, decreasing in difficulty with increased size). These were presented to the glabrous surface of the distal pad of the finger. Gratings were applied using a specially constructed apparatus designed to allow contact between the grating and the participant’s fingertip with constant pressure and position (Figure 2A). The gratings were applied for approximately 1 s per presentation, with an interstimulus interval of approximately 2 to 3 s.

On each trial of the testing task, the experimenter would present one of the seven testing gratings to the participant’s fingertip, with the grooves oriented either parallel or perpendicular with respect to the medial-proximal axis of the participant’s finger. Participants were asked to respond using a two-alternative forced choice (2AFC) whether the dome was parallel (“down”) or perpendicular (“across”; see Figure 2C).

In Blocks 1 and 2, each of the seven gratings was presented in a 10-trial block (in random order). Subsequently, the computer selected four grating sizes for additional data collection in a final block. These four gratings were selected to fall within the “interval of uncertainty” of the psychometric function. That is, we targeted gratings with variable accuracy. This was done by excluding any gratings that produced 100% correct performance from the selection range (if applicable). If no gratings produced 100% accuracy, then any with 90% accuracy were excluded. We then selected randomly from the gratings that were left. Thus, for each finger, three from seven gratings were presented in one block (10 trials), and four were presented in two blocks (20 trials), resulting in 110 trials in total per finger/test. Accuracy feedback (0%–100%) was provided over headphones randomly, on approximately one third of the blocks. Overall, the testing sessions lasted approximately 1 hr, with short intrablock breaks.

### Sensitivity Check

We used a short sensitivity check to determine whether we achieved a significant reduction in information from slowly adapting mechanoreceptors mediating the performance of orientation discrimination (Johnson, 2001; Van Boven & Johnson, 1994). As in the testing procedure, responses were 2AFC, so chance performance corresponded with 50% accuracy. The sensitivity check used an abbreviated version (∼2 min) of the testing task, that is, only 10 presentations of the largest grating (3.5 mm). Effective reduction in perception was achieved: The Sham + Train group demonstrated 100% accuracy (*SEM* = 0; i.e., all participants performed with complete accuracy), the Block-only group performed at chance (54.09% accuracy; *SEM* = 3.78; accuracy nonsignificantly different from chance, as demonstrated by a one-sample *t* test comparing accuracy with 50% chance, *p* = .437), as did the Block + Train group (52.27% accuracy; *SEM* = 5.00; also nonsignificantly different from chance, *p* = .615). Independent-samples *t* tests indicated there was no difference in accuracy between the blocked groups (*p* = .967) and that both blocked groups had significantly lower accuracy than the sham group (*p* < .001).

### Minimal Training Task

Training sessions were used to improve perception of tactile grating orientation. Although this task was originally considered to be resistant to training effects (Johnson & Phillips, 1981; Van Boven & Johnson, 1994), later studies have shown this task to robustly produce tactile perceptual learning following training (Dempsey-Jones et al., 2016; Harrar et al., 2014; Sathian & Zangaladze, 1997). The trained finger was the middle finger of the right hand. The task used for training differed from the testing task, to encourage participants to learn tactile features of the stimuli rather than task requirements. On each trial, the grating was presented twice to the trained finger (using the same apparatus and timing as in testing). Participants were asked to report whether both presentations were oriented in the same direction (e.g., both down) or in different directions (e.g., down-across; also 2AFC, see Figure 2C). Feedback on accuracy was provided over headphones trial-by-trial to maximize learning (“correct”/“incorrect”).

The gratings used for training were selected for each participant to be two above and two below that individual’s perceptual threshold, as determined at baseline (Dempsey-Jones et al., 2016; Harrar et al., 2014). A larger range of (*n* = 10) grating spacings was used for training to allow closer matching to the participant’s threshold (sizes were the same as the testing stimuli, with the addition of 0.75, 2.0, and 3.0).

Training consisted of six blocks (four grating sizes/block; 12 trials/grating—in which one trial consisted of two presentations of the grating stimuli; see section: Testing Task). There were two blocks of training, on the first and second days, respectively. One training session lasted approximately 45 min, with short intrablock breaks). We used a short training, as we aimed for minimal learning in order to avoid potential training ceiling effects when examining the added benefits of deafferentation (i.e., allowing any additional benefit of Block + Training to reveal itself compared with Sham + Training).

### Determining Perceptual Thresholds

Tactile psychophysical thresholds for each finger and testing session were determined by plotting accuracy as a function of grating size across all levels of stimulus difficulty. The data were fitted with a Weibull curve using a least-squares function in MATLAB (two free parameters; gamma and lambda set at .05 and 0, respectively). The threshold for this psychometric function was interpolated from the grating size estimated to yield 82% accuracy.

Baseline thresholds for our sample were quantitatively and qualitatively similar to those collected from several independent samples that we have previously published using the same testing method and stimuli (Dempsey-Jones et al., 2016; Harrar et al., 2014), although note that raw thresholds were higher than some previously published studies likely as a result of the use of the method of constant stimuli for grating difficulty presentation as opposed to a descending staircase that produces lower absolute thresholds (see online supplemental materials, Part 2 [Table S1] for raw thresholds and Part 3 for further discussion).

### Goodness of Fit of the Psychometric Functions

In 4.8% of the cases (42 of 864 cases: 6 fingers × 4 sessions × 36 participants = 864), the algorithm was unable to fit a curve to the data using the specified parameters. This occurred because the data to be fitted violated the assumptions of the Weibull curve beyond the defined tolerance limits (e.g., there was not a reasonable incremental increase in accuracy with increasing stimulus size). For these 42 cases, we attempted to refit the curve by removing a single outlying data point (i.e., accuracy score for a single grating) if said point was deemed to be an outlier. To identify outlier data points, we plotted all data for all participants and conditions onto a grand mean plot and removed a data point if it fell outside ±3 standard deviations of the grand mean and was thus considered an outlier. Removing single problematic data points allowed us to fit a curve to the remaining data in all but 16 functions (1.9% of all original cases) that had to be excluded from further analysis.

Over the whole data set, the psychometric functions predicted the data with good accuracy (average *R*^2^ = .72, *SEM* = .01). However, some individual psychometric functions showed very poor fits to the data. We therefore removed functions with low *R*^2^ (*R*^2^ < .15; seven cases from the remaining 848, leaving 841 cases), because values below this level represent very low-fitting success considering the percentage of variance in the data explained by the psychometric function fit (Swanson & Birch, 1992). That is, these data points were removed not to improve model convergence in the generalized estimating equation (GEE) but rather because they did not represent valid thresholds as produced by the psychophysical thresholding procedure (GEE model convergence was good, see corrected quasi-likelihood under independence model criterion [QICC] values in Tables 1 and 2 in the Results section). In the interest of reliability, an additional analysis was performed on the full data set (without excluding these cases). This produced the same pattern of results as reported below.[Table-anchor tbl1][Table-anchor tbl2]

Supporting the stability of our thresholds over time, we found that there was no difference in goodness of fit (*R*^2^) across the four testing sessions for any of the three groups (.200 < *p* > .744; i.e., curve fitting was equally successful). High consistency in mean and *SEM* values between our study and previous studies (from our laboratory and externally) also support the stability of our data and fitting procedures (see online supplemental materials, Part 3).

### Normalization of Data

Data were baseline normalized to best reflect change over sessions for each finger, independent of minor baseline threshold differences between fingers that were irrelevant to the results of interest (Dempsey-Jones et al., 2016; Harrar et al., 2014; Vega-Bermudez & Johnson, 2001; note that no baseline differences were found between groups for any finger, .137 < *p* > .438). Normalization was achieved by subtracting the baseline threshold from subsequent thresholds (individually for each participant and finger). Raw data are presented and visualized in Part 2 of the online supplemental materials; normalized data with individual case (single participant) data are also available in the online supplemental materials (Part 4; and online at http://www.ucl.ac.uk/icn/research/supps/dempseyjones).

In all visualizations, we present actual means rather than estimated marginal means (generated by the statistical analyses) to best represent the actual data values and variability.

### Analyses

GEE analyses were selected to examine the current data set because such methods are better able to account for the interdependence between data compared with ANOVA methods (by allowing explicit specification of the working correlation matrix between dependent variables), thus providing a better fitting model (Ballinger, 2004; though note that we replicate our central results with ANOVA methods in the online supplemental materials, Part 5, for comparability). Additionally, the GEE approach is also able to deal with missing data points (e.g., from curves that did not generate).

The threshold data were normally distributed: Thresholds for all six fingers at all four sessions were assessed for normality using a Kolmogorov–Smirnov test, with 23 of 24 thresholds (6 fingers × 4 sessions) found not to be different from a normal distribution (all *p*s > .05, aside from the right index finger in the online session; all 24 were *p* > .05 when corrections were applied for multiple comparisons).

GEE analyses were conducted using a linear scale model. This model was chosen for parsimony, as we had no a priori reason to specify a higher order or more complex model. The working correlation matrix was set as exchangeable, rather than independent, to maximize the model fit (reflected by the QICC). Session was coded as an ordinal factor (not continuous, as there were not continuous gaps between sessions, allowing us to test deafferentation effects at specific critical times postintervention; see experimental timeline in Figure 2). The GEEs were implemented with IBM SPSS Statistics, Version 22.0 (IBM, Armonk, NY).

For all analyses, results (χ^2^ and *p* values) are presented for major comparisons in the text, and comparisons not relevant to hypotheses and other lower order effects are presented in Tables 1 and 2.

### Comparisons

The first “parent” GEEs compared all fingers at all sessions—either within or between groups depending on the test—to determine whether there was any difference in the way the six fingers change over sessions and to justify our follow-up analyses. To ensure these interactions were not driven by changes in threshold caused by ongoing anesthesia (i.e., numbing of the right index finger in blocked groups at the online session), we repeated any comparison including such data with these values removed. There was no change in the pattern of results, all interactions remained significant (see Tables 1–2 in the Results section; comparisons repeated in this way are marked with a subscript a). To avoid this issue and enhance ease of interpretation, for our hypothesis-driven follow-up analyses, we removed the online session data if the (injected) right index finger was being compared—looking then at the offline and retention sessions only. Further, because these follow-up analyses only used a subset of fingers at a time, we covaried out the raw baseline threshold to account for any interfinger differences that could affect interpretation of our results (Van Breukelen, 2006; Vickers, 2001), unlike in the parent GEEs, for which this is not necessary, as finger is balanced across hands, and thus main effects of finger are even. These follow-up analyses were conducted separately per session to explore how changes varied over time, and were thus Bonferroni corrected for multiple comparisons.

Here, we include our hypothesis-driven analyses only. These tests compare particular fingers from particular groups at a time based on a priori predictions (e.g., comparing the index and ring fingers of the Block + Train group vs. the Sham + Train group to investigate for enhanced learning transfer). In the interest of completeness and transparency, we have therefore included a data-driven, exploratory analysis of learning in *all* fingers, for *all* groups in the online supplemental materials (in the Results section, Part 5). These data-driven analyses provide a converging picture of results to the hypothesis-driven tests (see Discussion).

## Results

### Direct Effects of Deafferentation: Selective Learning in the Deafferentation-Adjacent Finger (Block-Only Group)

We wished to investigate whether administration of anesthetic block to the right index finger altered perceptual thresholds of the six tested fingers over sessions. Specifically, we predicted selective improvements on the deafferentation-adjacent finger—with no, or significantly reduced, perceptual change on the other fingers, as some nonselective, generalized improvement may be seen across all fingers because of repeated tactile testing alone (Dempsey-Jones et al., 2016; see online supplemental materials, Part 1) or limited deafferentation related change in the nonadjacent fingers (see Discussion).

**Figure 3 fig3:**
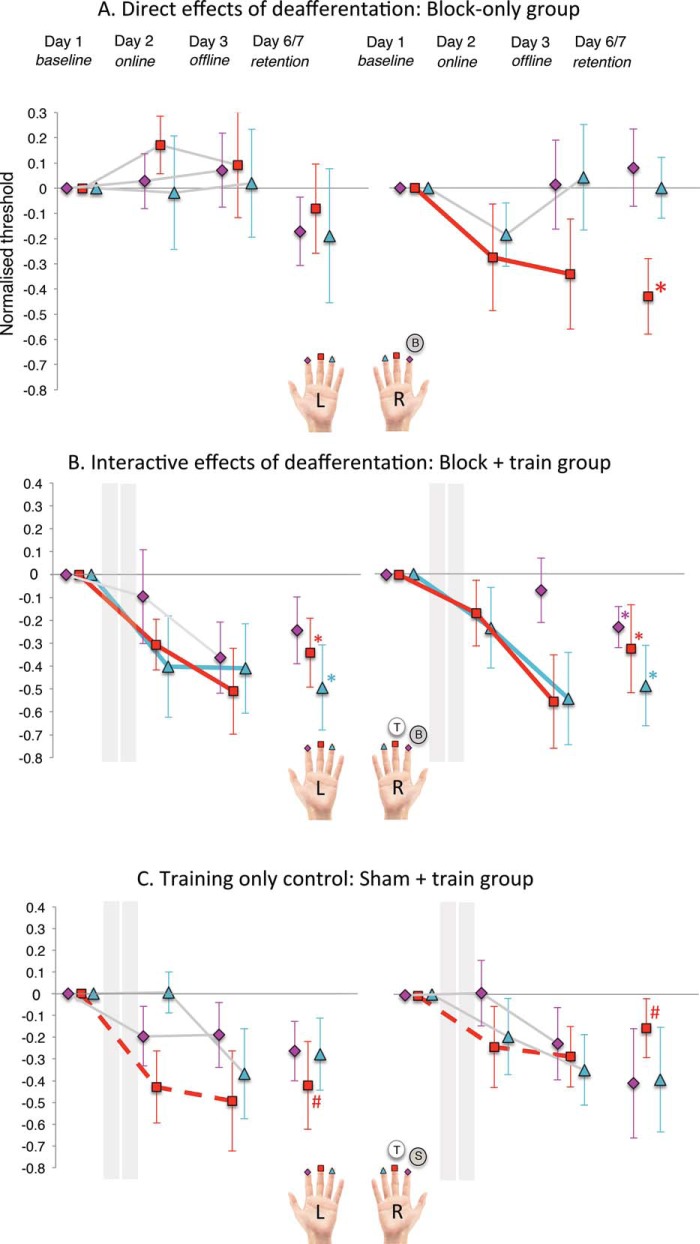
Change of tactile sensory thresholds over testing sessions in the three groups: (A) Block only (i.e., “direct” effects of deafferentation; top panel), (B) Block + Train (i.e., “interactive” effects of deafferentation; middle panel), and (C) Sham + Train (training-only control; bottom panel). Data are baseline normalized values (threshold minus baseline). See Part 2 of the online supplemental materials for raw data, and Part 3 for individual participant data (one point per condition/participant). Actual means are used (not estimated marginal means from the generalized estimating equation). Zero represents baseline perceptual threshold, and decreases from zero represent improved perception (negative numbers). Fingers that changed significantly in threshold over session, that is, that showed a significant main effect of session (see Table S3 of the online supplemental materials) are marked with an asterisk (e.g., the right middle finger of the Block-only group) and a block colored line. Fingers showing trending change are marked with a hashtag (#) and a dashed line. On the hand “legend,” fingers marked with a circle and “B” denote a blocked finger, those marked with “S” denote a sham-blocked finger, and the circle marked “T” denotes a trained finger (if applicable). Please note that for the blocked groups, the threshold for the right index finger is not represented for the period during which this finger was anesthetized (i.e., at the online test). Within-participants error bars are displayed here (Cousineau, 2005).

We found that there was indeed a difference in the way the fingers of the Block-only group changed in threshold over time. This was revealed by a 6 × 3 within-participants GEE analysis with factors Finger (left/right index, middle, ring) and Session (online, offline, retention) that produced a significant interaction of Finger × Session, χ^2^(10) = 111.41, *p* < .001. Lower order chi-square and *p* values in Table 1A; also see Figure 3 for visualizations. Note that comparing all six fingers in a 6 × 2 GEE returned only a trending difference (*p* = .075): this may indicate a loss of power resulting from the removal of data or it may suggest that selectivity of deafferentation gains may not be complete (see Discussion).[Fig-anchor fig3]

Next, we wished to directly contrast changes in the deafferentation-adjacent finger and the remaining five fingers of the hand—to determine whether gains were significantly larger for the right index finger compared with the other fingers, indicating relative selectivity. To do so, we collapsed over these five fingers to create an average threshold. Collapsing over fingers was deemed appropriate given that, critically, these five fingers changed in the same way over time (i.e., there was a nonsignificant interaction of Finger × Session, *p* = .167; see Table 1, column B).

As predicted, we found that there were greater perceptual gains in the deafferentation-adjacent finger than in the remaining five fingers of the hand. This difference, however, reduced by the long-term testing session (3–4 days postintervention). This was indicated by a within-participants GEE performed for each session with one factor, Finger (deafferentation-adjacent, average of remaining five). This produced a main effect of finger that was significant at the offline session (*p* = .003) but reduced to a trend at the long-term retention test (at Bonferroni corrected α = .025, *p* = .061; see Table 1, columns Ci and Cii). Descriptive statistics for the offline session indicated that the direction of this main effect was as expected, with greater learning decreases seen in the deafferentation-adjacent finger (*M* = −0.37, *SEM* = .23) than the remaining fingers (*M* = 0.23, *SEM* = .11). Results are presented for individual participants (one data point per condition/participant) in Part 4 of the online supplemental materials.

### Interactive Effects of Deafferentation: Enhancement of Learning Transfer in the Block + Train Versus Sham + Train Group

We next wished to explore the interactive effects of deafferentation and training on perception. To do so, we compared perceptual changes over session in the Block + Train group versus the Sham + Train group.

As predicted, we found that deafferentation altered training-related learning gains compared with training alone. This was revealed by a 6 × 3 × 2 mixed GEE analysis with within-participants factors of Finger (left/right index, middle, ring) and Session (online, offline, retention) and a between-participants factor of Group (Block + Train, Sham + Train), which produced a significant Finger × Session × Group interaction, χ^2^(10) = 38.42, *p* < .001 (see Table 2, column A for lower order χ^2^ and *p* values, and Figure 3 for visualizations).

We then wished to explore whether, consistent with our predictions, deafferentation caused enhanced transfer of tactile perceptual learning. Specifically, did deafferentation enhance transfer to the index and ring fingers of the left (untrained) hand—as these are fingers that do not normally show gains from learning transfer (see introduction)?

Consistent with our hypothesis, we showed that there was more learning in the left index and ring fingers in the Block + Train group compared with the Sham + Train group, but this effect had also reduced by the final (long-term) testing session. This was revealed by a mixed 2 × 2 GEE performed for each session with the factors Finger (left index, left ring) and Group (Block + Train, Sham + Train). This revealed that there was a significant main effect of group for the offline session (at α = .025, *p* = .018), but this became nonsignificant by the long-term retention test (*p* = .425; see Table 2, columns Bi and Bii). Looking at the descriptive statistics for the offline session, we saw that the direction of the main effect was as predicted—with greater threshold decreases (improved perception) in the Block + Train group (averaged across fingers, *M* = −0.39, *SEM* = .13) than the Sham + Train group (also averaged, *M* = −0.27, *SEM* = .13).

### Dissociation of Direct and Interactive Effects: Block-Only Versus Block + Train Group

Finally, we wished to demonstrate that the direct and interactive effects of deafferentation were truly dissociable in the pattern of perceptual gains they produce. As predicted, we found that the thresholds of the Block-only and Block + Train groups did change differently over fingers and sessions. This indicated that sensory improvements in the Block + Train group were attributable to *both* the effects of training and the block (not the block alone), and these interactive effects were thus statistically distinguishable from the direct effects of the block alone seen in the Block-only group.

This was revealed by the results of a 6 × 3 × 2 mixed GEE analysis with within-participants factors of Finger (left/right index, middle, ring) and Session (online, offline, retention) and a between-participants factor of Group (block only, Block + Train) that produced a significant Finger × Session × Group interaction, χ^2^(10) = 26.29, *p* = .003 (see Table 2, column C for lower order χ^2^ and *p* values, and Figure 3 for visualizations).

We then examined whether the difference between these two groups aligned with our specific hypotheses. As discussed in the introduction, we had predicted threshold gains for the deafferentation-adjacent (right middle) finger in both the block-only and Block + Train group. However, we predicted that gains would be largely selective to this finger in the Block-only group. In contrast, we expected there would be widespread gains across the hand in (up to) all five remaining fingers in the Block + Train group—resulting from the interaction of training and deafferentation. As predicted, we found greater learning gains across these five fingers in the Block + Train group compared with the same fingers of the Block-only group. As with previous results, however, this effect reduced by the long-term test.

This was revealed by a 5 × 2 mixed GEE with the factors Finger (left/right index, left middle and left/right ring) and Group (block only, block + train) conducted for both the offline and retention sessions. These analyses revealed that the main effect of group was significant for the offline test (at α = .025, *p* = .006) but reduced to a trend by the long-term retention test (*p* = .062; see Table 2, columns Di and Dii). Descriptive statistics at the online test indicated that, consistent with expectations, there was greater threshold drop (and thus, improved perception) in fingers of the Block + Train group (averaged over fingers; *M* = −0.38, *SEM* = .08) than the block-only group (also averaged; *M* = 0.05, *SEM* = .09). Group did not interact with finger at the offline or retention tests (.841 and .406, respectively), indicating all five fingers were nonsignificantly different in threshold at either session, that is, there was consistency in tactile perception between fingers at both tests (also see Table 2, column D).

In addition to our hypothesis-driven analyses (above), we also performed data-driven (within-groups) analyses on each group separately to investigate in more detail how *each* finger changed individually across sessions for *each* group. Because of the exploratory and descriptive nature of these results, we report them in the online supplemental materials (Part 6). The results of these data-driven analyses reflected the hypothesis-driven tests. Finally, we present a description of the point at which significant changes in threshold occurred for each finger (for each group), termed the *time to learn* analysis (see online supplemental materials, Part 7).

## Discussion

It is now widely supported that sensory input loss causes changes in brain organization. In contrast, whether and how reorganization functionally shape perception has remained unclear (Makin & Bensmaia, 2017). Perceptual gains could be triggered by the direct effects of sensory loss (recruitment of deafferented cortex; Merzenich, Kaas, Wall, Nelson, et al., 1983). They could also occur by facilitation of concurrent sensory input that co-occurred with the deafferentation, for example, training (Muellbacher et al., 2002; Rosen et al., 2006; Shibata et al., 2012; Ziemann et al., 2001), or changes in behavior to compensate for deafferentation, for example, exploration behavior using the deprived sensory organ, after the sense of touch had been restored (Polley, Chen-Bee, & Frostig, 1999). Upper-limb amputees present a classic example of this duality, as amputation causes both input loss and dramatic behavioral change (Hahamy et al., 2015, 2017; Makin et al., 2013; see also Kupers & Ptito, 2014).

Here, we aimed to disentangle this ambiguity by determining the relative contributions of deafferentation and concomitant sensory training on perceptual gains. Using psychophysical measures, we found that temporary finger deafferentation directly enhanced tactile perception of the deafferentation-adjacent finger. We also demonstrated that sensory block concurrent to tactile training caused widespread transfer of learning to untrained fingers—beyond what was seen with sham block and beyond the normal topographic spread of tactile learning (Dempsey-Jones et al., 2016; Harrar et al., 2014). Our results suggest that deafferentation enhances perception both directly and interactively (by boosting the effects of sensory training), resulting in distinct profiles of sensory gains. This dissociation expands possibilities for the use of deafferentation for boosting sensory perception or promoting rehabilitation training following sensory insult or injury.

### How Could Deafferentation Directly Impact Tactile Perception?

What mechanisms might support selective gains in sensory thresholds in the deafferentation-adjacent finger? Cortical and subcortical deafferentation-related changes are likely inherently linked (Kambi et al., 2014). Here, we focus our discussion on documented changes in primary somatosensory cortex (SI), which have been studied most extensively, allowing a more comprehensive mechanistic understanding of deafferentation-related physiological changes.

SI reorganization after deafferentation is largely driven by alterations of the excitation–inhibition balance and Hebbian plasticity processes. Merzenich and colleagues revealed that several months after finger amputation (or median nerve transection), the cortical territory previously representing the deafferented finger(s) was subsumed by the adjacent fingers (Merzenich, Kaas, Wall, Sur, et al., 1983; Merzenich et al., 1984; see also Pons et al., 1991; and see Feldman & Brecht, 2005, for results in rodents). In rats, Faggin and colleagues (1997) showed deafferentation-related changes across the somatosensory system occurring almost immediately following anesthetic whisker block. The rapid time scale of these changes suggests that reorganization is supported by the unmasking of preexisting connections (“silent cells”) between adjacent cortical areas (Margolis et al., 2012). Unmasking may occur because of disinhibition, which is known to be important in maintaining distinct borders between representations (SI: Jones, 1993; Paullus & Hickmott, 2011; M1: Jacobs & Donoghue, 1991). Thus, deafferentation causes near-immediate increases in processing resources for spared sensory inputs. Supporting this, training-related increases in cortical areal extent correlate with perceptual gains in tactile learning studies (Recanzone, Merzenich, Jenkins, Grajski, & Dinse, 1992), suggesting that deafferentation could cause similar gains by increasing cortical representations.

The selective gains we document on the right middle finger are unlikely to have occurred as a result of repeated exposure to our testing procedure. Although not shown here, we have previously demonstrated, in two independent samples, that testing the right and left index, middle, and ring fingers—using an identical protocol over multiple testing days—causes limited, but importantly, equivalent (i.e., nonselective) gains in perception for all six fingers (Dempsey-Jones et al., 2016; Harrar et al., 2014; full details in the online supplemental materials, Part 1).

The physiological literature suggests that although the majority of deafferentation effects occur for bodily locations directly adjacent to the deafferented zone, effects are not restricted to adjacent locations—with reduced changes being documented further afield (Merzenich et al., 1984) almost instantaneous to deafferentation (in whiskers, see Faggin et al., 1997). Although our a priori results and exploratory analyses (see the online supplemental materials, Results, Part 6) suggest selectivity of gains, selectivity may not be complete (see trend in the Block-only results). Thus, it may be that with longer deafferentation (e.g., over 2 hr, as here), we may see gains in fingers other than the deafferentation adjacent finger. Given the results of physiological studies (above), however, we expect effects to be most pronounced in the adjacent finger—regardless of deafferentation duration.

### How Could Deafferentation Interact With Training to Cause Learning Gains?

Our second key prediction was that deprivation can drive sensory gains by modulating the processing of sensory input concurrent to input loss (here, training effects), thereby resulting in a divergent pattern of gains for touch perception compared with deafferentation alone. More specifically, we predicted that deafferentation would cause training-related learning gains to transfer beyond the normal extent of topographic transfer. Previous studies using similar designs have demonstrated that learning transfer causes a specific and restricted pattern of learning gains, with transfer from the trained finger to the adjacent and homologous fingers alone (and not to other fingers outside these topographic relational categories; Dempsey-Jones et al., 2016; Harrar et al., 2014). Subsequently, we wished to determine whether we could expand this transfer boundary. We predicted that the interactive effect of training and deafferentation would result in transfer of learning to the index and ring fingers of the untrained hand (which typically do not learn under normal circumstances, i.e., no deafferentation). Consistent with our prediction, we found that the extent of learning transfer was greater in these fingers when training was coupled with sensory block compared with when coupled with sham block.

This boost in learning transfer may have resulted from the direct effects of deafferentation: For instance, invasion of the deafferented finger territory by the deafferentation-adjacent finger or fingers may have altered the pattern of learning transfer by changing topographic neighborhood relationships in the somatosensory system. In such a case, fingers could become “adjacent” after deafferentation where they were not before, thus modulating the way learning can transfer between fingers (Dempsey-Jones et al., 2016; Harrar et al., 2014; see the online supplemental materials, Part 8, for a discussion of the locus of tactile training effects within the somatosensory system). Given that training is also known to cause an increase in the areal extent of the trained skin surface (Detorakis & Rougier, 2014; Jenkins, Merzenich, Ochs, Allard, & Guíc-Robles, 1990; Xerri, Stern, & Merzenich, 1994; see Buonomano & Merzenich, 1998, for review) this could contribute to the way in which deafferentation and training interact to boost perception.

Training may also harness Hebbian plasticity processes triggered by deafferentation—causing enhanced training-related gains, for example, long-term depression (Allen, Celikel, & Feldman, 2003) and/or potentiation (Gambino & Holtmaat, 2012). This is consistent with previous rodent work suggesting deafferentation-related modulations of neuronal selectivity and tuning are altered by concurrent behavior (and the subsequent patterns of sensory input these behaviors cause; Polley et al., 1999). Indeed, increased training efficacy could account for the widespread transfer of learning and subsequent sensory gains across the hand that we show here (see Zeiler & Krakauer, 2013, for a similar theory of interactive effects of poststroke plasticity and learning).

Alternatively, enhanced transfer of learning gains following anesthetic block may reflect deafferentation-related alterations in RF properties. Tactile training has long been associated with changes in RF properties in SI (e.g., the shrinking and migration of RFs toward the trained area and [some] adjacent areas: Jenkins et al., 1990; Recanzone et al., 1992; modeled by Detorakis & Rougier, 2014). It has been suggested that RF overlap may critically drive the transfer of tactile learning (Harrar et al., 2014; also see Harris et al., 2001). Thus, the increased overlap of RFs representing the spared, neighboring fingers (Merzenich, Kaas, Wall, Nelson, et al., 1983) might facilitate enhanced learning transfer following deafferentation that we demonstrate here.

### Deafferentation and Experience-Dependent Plasticity

Although we demonstrate that direct and interactive effects of temporary deprivation are distinct in the patterns of sensory gains they produce, we believe these processes are likely supported by a related mechanism. We previously emphasized the role of habitual behavior in shaping SI organization and, subsequently, transfer patterns of tactile learning (Dempsey-Jones et al., 2016; see also Ejaz, Hamada, & Diedrichsen, 2015). Our current findings highlight the need to consider behavioral changes (especially with nondeafferented [spared] body parts) in understanding deafferentation related plasticity (Makin et al., 2013). Indeed, it is possible that undocumented behavioral changes subsequent to deafferentation could contribute to the “direct” sensory improvements we report. For example, because we did not restrict the movements of our participants in the Block-only group during and postdeafferentation, they may have increased reliance on their deafferentation-adjacent finger because of the altered state of their hand. Indeed, deafferentation could combine with hand use related to our testing or training protocols as well as naturalistic behavior in the experiment breaks and following cessation of testing (while residual deafferentation effects lingered). The use of the mouse to respond in our study, for instance, could have provided tactile feedback to the index and middle fingers (of both hands during testing, and the left hand during training, if applicable). This may have led to a reduction in tactile thresholds on these two fingers because of unintentional “training” (although this appears unlikely given nonsignificant tactile gains in the left/right index finger—used with the mouse—in either the Block-only or the Sham + Train groups).

Given the potential influence of undocumented tactile stimulation, we suggest that sensory improvements in the Block-only group could also have resulted, in part, from the interaction of deafferentation and sensory experience. It may therefore be more appropriate to term direct and interactive effects as *weakly interactive* and *strongly interactive* effects of deafferentation and training. This finding emphasizes the tight link between deprivation-driven and experience-dependent plasticity. In this way, our results complement those from studies of visual deprivation (e.g., Duffy & Mitchell, 2013; Lunghi, Emir, Morrone, & Bridge, 2015) and demonstrate that even transient somatosensory input loss can reset sensory pathways to a more plastic state.

## Supplementary Material

10.1037/xge0000514.supp

## Figures and Tables

**Table 1 tbl1:** Complete Statistical Details for the Within-Participants Generalized Estimating Equation (GEE) Analyses Presented in Text for the Block-Only Group

Comparison	A Group (1: block only) Finger (6: all) Session (3: all)^a^	B Group (1: block only) Finger (5: no R middle) Session (2: no online)	Ci Offline	Cii Retention
			Group (1: block only) Finger (2: R middle, av. remaining 5)	
Finger	χ^2^(5) = 11.67, *p* = .040*	χ^2^(4) = 6.66, *p* = .155	χ^2^(1) = 8.90, *p* = .003*	χ^2^(1) = 3.52, *p* = .061
Session	χ^2^(2) = 7.97, *p* = .019*	χ^2^(1) = 1.70, *p* = .193		
Group				
Finger × Session	χ^2^(10) = 111.41, *p* < .001*	χ^2^(4) = 6.47, *p* = .167		
Finger × Group				
Session × Group				
Finger × Session × Group				
QICC	128.52	73.52	17.67	11.76
*Note.* Columns A to C contain GEE analyses, (A) for all six fingers and three sessions, which indicates fingers change differently over finger and session; (B) analyses with the deafferentation-adjacent finger removed reveals the remaining fingers change in the same way over sessions (i.e., collapsing values over these fingers is appropriate; ‘R’ denotes ‘right’ in the heading); and (C) hypothesis-driven follow-up tests. This reveals significant differences between the trained finger vs. five remaining fingers (av. remaining 5) in the offline session. This corresponds to a main effect of Finger (Ci), reducing to a trend by the long-term retention sessions (Cii; see in-text for direction of this finger main effect and its interpretation). Follow-up GEEs in Column C were Bonferroni corrected for multiple comparisons (α = .25). QICC = corrected quasi-likelihood under independence model criterion. Shaded sections indicate critical interactions.				
^a^ Indicates this comparison was rerun without data for the injected finger while anaesthetic effects may have still been apparent (right index finger, online session); interaction remained significant at *p* < .05.				
* Indicates a significant difference *p* < .05.				

**Table 2 tbl2:** Complete Statistical Details for the Between-Participants Generalized Estimating Equation (GEE) Analyses Presented in Text for Between-Group Comparisons

Comparison	A Group (2: Block + Train, Sham + Train) Finger (6: all) Session (3: all)^a^	Bi Offline	Bii Retention	C Group (2: block only, Block + Train) Finger (6: all) Session (3: all)^a^	Di Offline	Dii Retention
		Group (2: Block + Train, Sham + Train) Finger (2: left index, left ring)			Group (2: block only, Block + Train) Finger (5: no R middle)	
Finger	χ^2^(5) = 19.53, *p* = .002*	χ^2^(1) = 29.11, *p* < .001*	χ^2^(1) = 9.43, *p* = .002*	χ^2^(5) = 18.85, *p* = .002*	χ^2^(4) = 17.61, *p* = .001*	χ^2^(4) = 32.75, *p* < .001*
Session	χ^2^(2) = 3.30, *p* = .192			χ^2^(2) = 2.33, *p* = .312		
Group	χ^2^(1) = .42, *p* = .519	χ^2^(1) = 5.56, *p* = .018*	χ^2^(1) = .64, *p* = .425	χ^2^(1) = 5.09, *p* = .024*	χ^2^(1) = 7.68, *p* = .006*	χ^2^(1) = 3.52, *p* = .062
Finger × Session	χ^2^(10) = 55.99, *p* < .001*			χ^2^(10) = 35.01, *p* < .001*		
Finger × Group	χ^2^(5) = 5.92, *p* = .314	χ^2^(1) = .42, *p* = .517	χ^2^(1) = .42, *p* = .515	χ^2^(5) = 4.07, *p* = .539	χ^2^(4) = 1.42, *p* = .841	χ^2^(4) = 3.99, *p* = .406
Session × Group	χ^2^(2) = 5.98, *p* = .050			χ^2^(2) = 7.45, *p* = .024*		
						
Finger × Session × Group	χ^2^(10) = 38.42, *p* < .001*			χ^2^(10) = 26.29, *p* = .003*		
QICC	230.04	24.45	24.41	244.85	72.20	63.42
*Note.* Columns A and B contain analyses presented for the trained groups, (A) for all six fingers and three sessions, revealing a difference in the way thresholds change over session for the fingers; and (B) hypothesis-driven follow-up tests between the left index/ring of the Block + Train vs. Sham + Train groups, which indicate a significant group effect (main effect of group) in the offline (Bi) but not long-term retention sessions (Bii; see in-text for direction of group main effect and its interpretation). Columns C and D contain analyses presented for the blocked groups, (C) for all six fingers and three sessions, also revealing a difference in threshold change over session between fingers; and (D) hypothesis-driven, follow-up tests between the five fingers tested (no deafferentation-adjacent finger) for the block only group vs. the Block + Train group, revealing a significant group effect (main effect of group) at the offline (Di) but not retention sessions (Dii; see in-text for direction of this group main effect and its interpretation). Follow-up GEEs in Columns B and D were Bonferroni corrected for multiple comparisons (α = .25). QICC = corrected quasi-likelihood under independence model criterion. Shaded sections indicate critical interactions.						
^a^ Indicates this comparison was rerun without data for the injected finger while anaesthetic effects may have still been apparent (right index finger, online session); interaction remained *p* < .05.						
* Indicates a significant difference at *p* = .05.						

**Figure 1 fig1:**
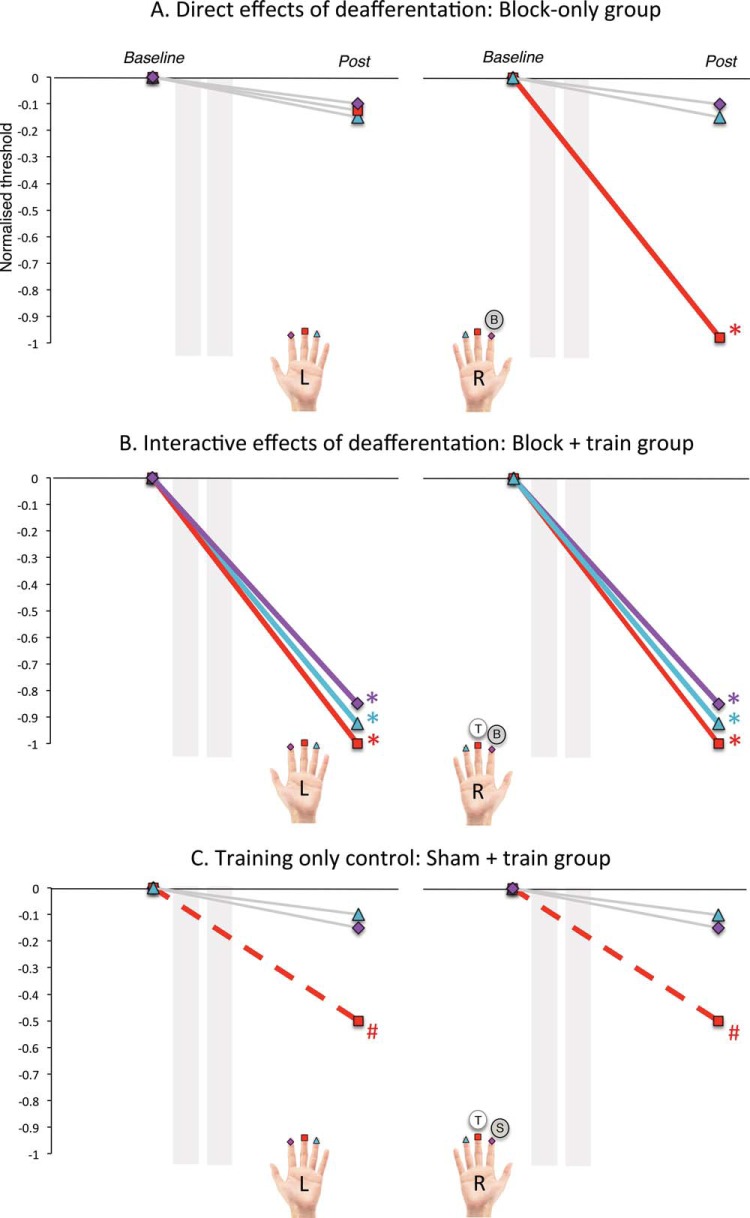
Prediction figure: schematic representation of hypothesized changes in touch perception from pre- to post-intervention in the three groups: (A) Block only (i.e., “direct” effects of deafferentation; top panel), (B) Block + Train (i.e., “interactive” effects of deafferentation; middle panel), and (C) Sham + Train (training-only control; bottom panel). Values are baseline normalized (threshold minus baseline); thus, zero represents baseline perceptual threshold, and threshold decreases from zero represent improved perception (negative numbers). We predicted (A) in the Block-only group, direct deafferentation effects would produce sensory gains that were mostly selective to the deafferentation-adjacent (right middle) finger; (B) in the Block + Train group, interactive effects would lead to gains that were much more widespread, that is, learning for (up to) all six tested fingers; and finally, (C) in the Sham + Train group, we predicted limited learning in the trained finger, with possible transfer of learning to the homologous finger, resulting from the effects of the minimal training paradigm alone. Fingers that were predicted to improve significantly are marked with an asterisk and a block-colored line (*); those that are expected to show some limited improvement that may not reach significance, with a hashtag and dashed line (#); fingers predicted not to change significantly are indicated by gray lines. On the hand “legend,” fingers marked with a circle and “B” denote a blocked finger, those marked with “S” denote a sham-blocked finger, and the circle marked “T” denotes a trained finger (if applicable).
