# Supplementary material for: Blocking Tactile Input to One Finger Using Anaesthetic Enhances Touch Perception and Learning in Other Fingers
Source: J Exp Psychol Gen. 2019 Apr;148(4):713–27. doi: 10.1037/xge0000514 (PMC6459089; doi:10.1037/xge0000514)
Supplement: Supplementary file 1 [file xge0000514SuppResMatclean.docx]

**Supplementary Materials**

**Part I. Supplementary Results: ‘Testing only’ control group, independent dataset**

In the current study we compare a deafferentation only group (‘block only’), a deafferentation and training group (‘block+train’), and a sham deafferentation and training control group (‘sham+train’). We did not include a ‘sham only’ condition, where participants underwent a protocol of repeated testing alongside two ‘sham’ blocks (akin to the ‘block only’ group). A major consideration in this decision was that we have previously run two studies examining the effects of repeated tactile testing alone on the tested fingers i.e., without training (a ‘testing only’ control: Harrar et al., 2014; Dempsey-Jones et al., 2016). In both previous studies, we have established that repeated testing alone does not cause selective change in touch thresholds for any one finger. This allows us to interpret the effects of the nerve block in the block only group indirectly. It also means repeated testing cannot explain isolated improvement of the right middle finger in the block only group. Given these replicated findings, a ‘sham only’ group would arguably not be additionally informative here.

Here, for the interest of readers, we provide further details of the analysis of one such ‘testing only’ control group from one of the aforementioned independent datasets (Dempsey-Jones et al., 2016). In this study, participants underwent an identical tactile testing procedure to that used in the current study (same stimuli, design and equipment). This testing, however, was carried out over a week, involving five tests (one in addition to the testing applied in the present study, i.e., even greater exposure to testing).

Analysis of this testing only control group showed that, as had been predicted, there was some limited improvement in threshold (as a result of repeated testing) but this improvement was not different between fingers. This was revealed by a within-participants ANOVA with factors Finger (right/ left index, middle, ring) and Session (1-5) that produced a significant main effect of testing Session (F (2,14) = 10.12, p = 0.003), a significant main effect of Finger (F (5,40) = 3.94, p = .005), but no interaction of Finger x Session (F (5,41) = 0.76, p = .758). See Figure 2C, Dempsey-Jones et al., 2016. This consistency across fingers supports the role of our intervention in causing selective change in the right middle finger in the block only group (also see similar results in Harrar et al., 2014).

**Part II. Table S1.** *Raw (non-normalised) data for all experimental groups, I. the block only group, II. The block+train group and III. The sham+train group. Mean values are shown in the left four columns and standard error of the mean (SEM) values in the right four columns. ‘block’ indicates a deafferented finger, ‘sham’ a sham-deafferented finger and ‘train’ a trained finger, where applicable.*

| I. Block only | Means | | | | | | | | SEM | | | | | | | |
| --- | --- | --- | --- | --- | --- | --- | --- | --- | --- | --- | --- | --- | --- | --- | --- | --- |
|  | **Baseline** | **Online** | | **Offline** | | | **Retention** | | **Baseline** | | **Online** | | **Offline** | | **Retention** | |
| **R index (block)** | 1.78 | 1.91 | | 1.79 | | | 1.87 | | 0.11 | | 0.24 | | 0.18 | | 0.19 | |
| **R middle** | 2.15 | 1.84 | | 1.77 | | | 1.67 | | 0.17 | | 0.17 | | 0.15 | | 0.15 | |
| **R ring** | 2.28 | 2.08 | | 2.33 | | | 2.28 | | 0.19 | | 0.11 | | 0.15 | | 0.12 | |
| **L ring** | 2.29 | 2.27 | | 2.31 | | | 2.10 | | 0.19 | | 0.19 | | 0.17 | | 0.21 | |
| **L middle** | 1.84 | 2.01 | | 1.93 | | | 1.76 | | 0.17 | | 0.14 | | 0.16 | | 0.13 | |
| **L index** | 1.79 | 1.82 | | 1.86 | | | 1.62 | | 0.13 | | 0.12 | | 0.20 | | 0.11 | |
|  |  |  | |  | | |  | |  | |  | |  | |  | |
| II. Block+train | Means | | | | | | | SEM | | | | | | | |  |
|  | **Baseline** | | **Online** | | **Offline** | **Retention** | | **Baseline** | | **Online** | | **Offline** | | **Retention** | |  |
| **R index (block)** | 1.67 | | 2.58 | | 1.60 | 1.44 | | 0.14 | | 0.24 | | 0.19 | | 0.15 | |  |
| **R middle (train)** | 1.97 | | 1.93 | | 1.56 | 1.78 | | 0.12 | | 0.15 | | 0.15 | | 0.23 | |  |
| **R ring** | 2.27 | | 2.01 | | 1.90 | 1.95 | | 0.17 | | 0.14 | | 0.22 | | 0.14 | |  |
| **L ring** | 2.52 | | 2.12 | | 2.11 | 2.02 | | 0.14 | | 0.21 | | 0.16 | | 0.13 | |  |
| **L middle** | 2.26 | | 1.92 | | 1.75 | 1.87 | | 0.16 | | 0.14 | | 0.13 | | 0.10 | |  |
| **L index** | 1.84 | | 1.65 | | 1.53 | 1.60 | | 0.11 | | 0.15 | | 0.23 | | 0.17 | |  |

|  |  |  |  |  | |  | |  | |  | |  | |  |
| --- | --- | --- | --- | --- | --- | --- | --- | --- | --- | --- | --- | --- | --- | --- |
| III. Sham+train | Means | | | | | | SEM | | | | | | | |
|  | **Baseline** | **Online** | **Offline** | | **Retention** | | **Baseline** | | **Online** | | **Offline** | | **Retention** | |
| **R index (sham)** | 2.04 | 2.05 | 1.82 | | 1.64 | | 0.19 | | 0.17 | | 0.20 | | 0.17 | |
| **R middle (train)** | 2.13 | 1.89 | 1.81 | | 1.98 | | 0.14 | | 0.20 | | 0.19 | | 0.20 | |
| **R ring** | 2.31 | 2.31 | 2.16 | | 2.08 | | 0.14 | | 0.14 | | 0.19 | | 0.18 | |
| **L ring** | 2.61 | 2.54 | 2.24 | | 2.33 | | 0.14 | | 0.11 | | 0.11 | | 0.12 | |
| **L middle** | 2.42 | 2.08 | 2.02 | | 2.09 | | 0.19 | | 0.12 | | 0.16 | | 0.12 | |
| **L index** | 1.96 | 1.76 | 1.76 | | 1.70 | | 0.19 | | 0.17 | | 0.14 | | 0.16 | |
|  |  |  |  | |  | |  | |  | |  | |  | |

**Part III. Grating orientation values against aspects of methodology**

With regard to the absolute values of these thresholds, procedures using descending staircases typically appear to produce lower threshold values (Sathian & Zangaladza, 1996; 1997; Van-Boven & Johnson, 1994) than those using randomised presentation of difficulty levels (e.g., the method of constant stimuli, as in the current study; also see Harrar et al., 2014; Dempsey-Jones et al., 2016). Studies using presentation orders midway between staircase and randomisation report midrange thresholds (e.g., adaptive or Bayesian staircase procedures: Peters et al., 2009; Wong et al., 2011; 2013).

Given the potential link between apparent threshold values (as well as other methodological/ participant factors like age: Stevens & Patterson, 1995; gender: Peters et al., 2009), ‘absolute’ threshold values may be difficult to interpret with respect to physiological factors such as innervation density (see discussion in Johnson and Phillips, 1981; Peters et al., 2009). For this reason, we prefer not to speculate on the physiological meaning of our absolute threshold values (e.g., whether our intervention pushes perception below a level set by peripheral receptors, into, for example, the range of hyperacuity: Sathian, Deshpande & Stilla, 2013) but interpret relative threshold change with respect to baseline alone.

**Table S2.** *We propose a relationship between method of grating presentation (re. order of difficulty levels) and the absolute value of thresholds: if descending staircases are coded as 0 (least randomised), adaptve/ Baysian staircases as 5 (mid-level randomisation) and method of contant stimuli as 10 (full randomisation), there is a trending correlation between index finger threshold and presentation method (Spearman’s rho = .498, p = .099, N = 12 studies). All threshold values represent grating resolution in millimetres (mm). Please note that these studies use different values for threshold interpolation and are thus only approximately comparable (differences of between 1-9%). Also note the studies with most participants (marked in green, N>20), have thresholds most similar to those of the current study.*

|  |  |  |  |  |  |  |  |  |  |  | ^ older sample (M = 39 yo) * our laboratory | | |
| --- | --- | --- | --- | --- | --- | --- | --- | --- | --- | --- | --- | --- | --- |
|  | Lowest threshold |  |  |  |  |  |  |  |  |  |  | | Highest threshold |
|  | Sathian & Zangaladze 1997 | Sathian & Zangaladze  1996 | Van Boven & Johnson, 1994 | Craig 1999 | Werhahn et al. 2002 | Vega-Bermudez & Johnson, 2001 | Peters et al. 2009 | Van Boven et al. 1991 | Wong et al. 2013 | Wong et al. 2011^ | Harrar et al. 2014* | Dempsey-Jones et al., 2016* | Dempsey-Jones et al.* |
|  | N = 8 | N = 7 | N = 14 | N = 7 | N = 15 | N = 8 | **N = 97** | N = 3 | N = 10 | **N = 55** | **N = 24** | **N = 21** | **N = 36** |
| D1 |  | 1.00 (.06) |  |  |  |  |  |  |  |  |  |  |  |
| D2 | ~0.80 (~.1)  average D2/D3 (most D2) | 0.89 (.05) | .98 (.12) | ~1.00 | 1.10 (.09) | ~1.23-1.26 | ~1.40-1.60 | ~1.70 | 1.60-1.80  average (9 on D2, 1 on D4) | 1.70-1.80 | 1.70 (.08) | 1.85 (.07) | 1.84 (.08) |
| D3 |  | 0.93 (.05) |  |  |  | 1.43-1.51 |  |  |  | 1.75 | 2.02 (.11) | 2.10 (.07) | 2.12 (.09) |
| D4 |  | 1.06 (.10) |  |  |  | 1.67-1.62 |  |  | As above | 1.9 | 2.19 (.12) | 2.39 (0.8) | 2.38 (.09) |
| D5 |  | 1.44 (.15) |  |  |  |  |  |  |  |  |  |  |  |
| Threshold interpolation value | d' = 1.34 (approx. 75% acc.) | 75% acc. | d’ = 1.35 (approx. 75% acc.) | 75% acc. | 75% acc. | 71% acc. | d' = 1 (76% acc.) | d' = 1.35 (~75% acc.) | d' = 1 (76% acc.) | d' = 1 (76% acc.) | 82% | 82% | 82% |
| Presenta-tion order | Descending Stair. | Descending Stair. | Unclear (may be descend-ing stair.) | Descend-ing Stair. | Descend-ing Stair. | Staircase with reversals | 50% Ss. stair. w. reversals; 50% Baysian algorithm | Unspec-ified | Baysian algorithm | Baysian algorithm | MOCS | MOCS | MOCS |
| Previous tactile testing or training? | Yes (various unrelated grating exps.) | No | No | Yes (4 prior grating exps.) | No | Yes (prior tactile discrim. task: 468 trials) | Unclear | Yes (2 un-related tactile exps.) | No | No | No | No | No |

**Part IV. Figure S1: Individual participant data (one data-point per participant/ condition).**

**Figure S1.** *Change of tactile sensory thresholds over testing sessions in the three groups: A. Block only (i.e., ‘direct’ effects of deafferentation, top panel), B. Block+train (i.e., ‘interactive’ effects of deafferentation, middle panel) and C. Sham+train (training only control, bottom panel). Data are baseline normalised values (threshold-baseline),see Supplementary Materials, part II-III for raw data. Actual means are used (not estimated marginal means from the GEE). Zero represents baseline perceptual threshold, and decreases from zero represent improved perception (negative numbers). Fingers that changed significantly in threshold over session, i.e., that showed a significant main effect of Session (see within-participants GEE Results in Table 3) are marked with an asterisk (e.g., the right middle finger of the block only group). On the hand ‘legend’, fingers marked with a circle and ‘B’ denote a blocked finger, those marked with ‘S’ denote a sham-blocked finger, and the circle marked ‘T’ denotes a trained finger (if applicable). Please note: for the blocked groups, the threshold for the right index finger is not represented for the period during which this finger was anaesthetised (i.e., at the online test). For raw data for all participants and conditions please see: <http://www.ucl.ac.uk/icn/research/supps/dempseyjones>.*

**Part V. Supplementary results: Replication of our central (GEE) results using analysis of variance analysis (ANOVA) methods**

Here we replicate our key, hypothesis-driven results using ANOVA methods for comparability with previous studies that utilise this method of analysis. However, please note that, statistically, the GEE is a more appropriate method for the analysis of interdependent data (e.g., multiple fingers/ sessions per participant), as compared to repeated-measures ANOVA (Ballinger, 2004; see further discussion in the main text).

One particular benefit of the GEE framework is the ability to deal with missing cases. The GEE uses modelling of within- and between-participants variance to predict missing case values and, subsequently, does not require them to be filled using an arbitrary criterion. For our ANOVA analysis we did not replace empty cases in our dataset e.g., with averages created within groups or participants, resulting in a smaller sample size per comparison/ test.

In the main text, we hypothesise that deafferentation concurrent to training (block+train) enhances training effects by increasing the transfer of tactile learning. This transfer of learning to untrained fingers is predicted to be greater than that resulting from training effects alone (i.e., greater transfer in the block+train group versus the sham+train group).

To support these predictions, we tested whether the block+train group showed more learning in the index and ring fingers of the left (untrained) hand than the same fingers of the sham+train group. These fingers do not normally show gains from learning transfer following training alone (see data-driven results for the sham+train group, and previous literature: Dempsey-Jones et al., 2016; Harrar et al., 2014). The results supported our prediction, as revealed by a mixed GEE comparing learning gains on these two fingers (left index/ ring) between the block+train and sham+train groups.

Subsequently, we have now repeated this comparison with a mixed ANOVA, which revealed that the pattern of results held. The factors in this ANOVA were Finger (2 levels: left index, left ring), Session (3 levels: online, offline, retention), and Group (2 levels: block+train, sham+train). As in the main text, baseline normalised values were used, with the baseline covaried out (see van Breukelen, 2005; Vickers, 2001).

Supporting our GEE results, there was a significant main effect of Group, F(1,15) = 5.39, p = .035 η__^^ = .26. Also consistent with the GEE, no other main effects or interactions were significant (.269 < p > .968; please see Figure 3 in the main text for visualisation). As the GEE revealed no main effect or interaction with finger, gains were consistent between the two fingers for both groups.

As our second main hypothesis, we predicted that learning gains in the block+train group were indeed due to the interaction of training *and* deafferentation, rather than simply to deafferentation alone. Critically, we demonstrated differences between the block+train group and the block only group that supported this prediction.

More specifically, to reveal this result we examined whether tactile acuity was greater for the fingers of the block+train group, as compared to the block only group (we excluded the deafferentation adjacent finger, which was predicted to learn in both block groups). Using a mixed GEE we demonstrated widespread and equivalent gains across all fingers as a result of training and deafferentation – with more restricted gains from deafferentation alone (though these were also equivalent across fingers). That is, there was greater learning in the five fingers (all except the right middle) for the block+train group, as compared to the block only group – and learning was the same between all fingers, within either group.

We repeated this comparison with a mixed ANOVA, which produced the same pattern of results. The factors of this ANOVA were Finger (5 levels: left/ right index, left middle, left/ right ring), Group (2 levels: block+train; block only), Session (2 levels: offline, retention); other details consistent with the ANOVA above, and main text.

Supporting the GEE results, we found a significant main effect of Group, F(1,17) =5.71, p = .029, η__^^ = .25 – where there were greater gains in the block+train group than the block only group. Also as in the main text GEE analysis, no other main effects or interactions were significant (.210 < p > .936). Thus gains were consistent across fingers, for both groups.

**Part VI. Data-driven (hypothesis-free) analysis – Statistical assessment of significant learning gains in each finger, separately for each group**

In addition to our hypothesis driven analyses (above), we also performed data-driven (within-groups) analyses on each group separately to investigate in more detail how each finger changed across sessions in the three groups. This was because our hypothesis-driven results compared specific subsets of fingers and groups. Consequently, this did not provide a complete picture of whether any one finger (within any one group) showed significant learning gains.

We, therefore, performed one GEE per group and interpreted the resulting main effect(s) of Session for each finger (Wald Chi-Square test), i.e., was there a change in threshold across the four sessions, for one finger at a time. As these final tests were exploratory and descriptive in nature, an uncorrected p value was used, results are presented in Table 3 (though summarised in text below) and we draw limited interpretations from the outcomes. These comparisons used raw (non-normalised data) to best assess change from baseline over sessions (as opposed to normalised data which would allow only comparisons of difference scores, i.e., reflecting the amount of *change* per session). For interest, we also characterise the timeline of learning for the individual fingers using a further exploratory “*time to learn”* analysis (i.e., at what session do learning gains occur; see also Dempsey-Jones et al., 2016). However, since these timing results do not directly relate to our main hypotheses, they will not be presented in the main text (please see Supplementary Materials, part VI for details).

*Block only group*

In the block only group, our data-driven comparisons provided converging support for the selectivity of learning gains of the deafferentation-adjacent finger – seen in the hypothesis-driven tests. Selectivity was revealed by a significant main effect of Session for the deafferentation-adjacent finger alone (p = .041; see Table 3A for statistics regarding non-significant change in the remaining fingers).

*Block+train group*

Looking at the block+train group, we found that there was learning across almost all fingers of both hands. This was revealed by a significant main effect of Session for five of the six fingers (.001 < p > .049): all fingers except for the left index finger that showed non-significant perceptual gains over sessions (p = .252; see Table 3B). It, therefore, remains unclear whether interactive effects of deafferentation and training are somewhat finger-selective, or whether they are global (i.e., to all fingers) and we are simply underpowered here to reveal significant perceptual changes (vs. baseline) in the left index finger. While the left index finger showed non-significant change from baseline, the previous analysis still holds that there are significantly *more* gains on this finger for the block+train group vs. the sham+train group, still supporting enhanced transfer.

*Sham+train group*

In the sham+train group, we found evidence of small, but non-significant improvements in the trained and homologous fingers (p = .099 and p = .055; see Table 3C) – consistent with our prediction of minimal learning and learning transfer in this group (see Introduction). No fingers, however, showed significant learning gains.

**Table S3.** *Generalised Estimating Equation (GEE) analyses for the exploratory analyses. The top rows contains comparisons of all six fingers and four sessions. These tests indicate that there were indeed differences in the way fingers changed over sessions (i.e., significant Finger x Session interactions), for all groups. The bottom row demonstrates which individual fingers changed significantly in threshold across the course of testing (i.e., which showed a significant improvement in threshold from baseline; as reflected by a significant main effect of Session). Given the data-driven nature of these results, tests are uncorrected for multiple comparisons and limited conclusions are drawn from the outcomes.
~ indicates this comparison was re-run without data for the injected finger while anaesthetic effects may have still been apparent (right index finger, online session): interaction remained p < .05.*

|  |  | **Difference scores** |  |
| --- | --- | --- | --- |
|  | **A** | **B** | **B** |
|  | Group (1: Block only),  Finger (6: all), Session (3: all)~ | Group (1: Block+train), Finger (6: all),  Session (3: all)~ | Group (1: Sham+train), Finger (6: all), Session (3: all)~ |
| Finger | χ^2^ (5) = 250.83, p < .001** | χ^2^ (5) = 25.33, p < .001** | χ^2^ (5) = 44.40, p < .001** |
| Session | χ^2^ (3) = 3.05, p = .384 | χ^2^ (3) = 29.24, p < .001** | χ^2^ (3) = 46.13, p = .001** |
| Finger x Session | χ^2^ (11) = 128.70, p < .001** | χ^2^ (11) = 440.51, p < .001** | χ^2^ (11) = 138.90, p < .001** |
| **QICC** | **130.21** | **126.75** | **127.41** |

|  |  |  |  |
| --- | --- | --- | --- |
|  | **Raw scores (data-driven comparisons)** | | |
|  | **F** | **D** | **E** |
| R index | χ^2^ (3) = 0.57, p = .902 | χ^2^ (3) = 10.10, p < .001** | χ^2^ (3) = 4.32, p = .229 |
| R middle | χ^2^ (3) = 8.27, p = .041* | χ^2^ (3) = 10.09, p = .018* | χ^2^ (3) = 6.28, p = .099^#^ |
| R ring | χ^2^ (3) = 4.24, p = .237 | χ^2^ (3) = 7.88, p = .049* | χ^2^ (3) = 2.73, p = .436 |
| L ring | χ^2^ (3) = 1.31, p = .726 | χ^2^ (3) = 9.27, p = .026* | χ^2^ (3) = 5.95, p = .114 |
| L middle | χ^2^ (3) = 3.75, p = .290 | χ^2^ (3) = 11.05, p = .011* | χ^2^ (3) = 7.61, p = .055^#^ |
| L index | χ^2^ (3) = 6.19, p = .103 | χ^2^ (3) = 4.09, p = .252 | χ^2^ (3) = 4.83, p = .185 |

**Part VII. Supplementary Results: The timeline of learning gains**

To follow up from the within-participants Generalised Estimating Equation (GEE) analyses presented in the main text, and characterise the timeline of learning for the individual fingers, we used an exploratory “*time to learn”* analysis (see Dempsey Jones et al. 2016). This analysis determines how long it took for each finger to significantly improve in threshold with respect to its baseline. Paired-sample *t*-tests were used to compare the baseline threshold to the threshold of the next session. If this was non-significant, the subsequent session was compared to baseline, until a significant difference was identified. Given the descriptive nature of this analysis, an uncorrected alpha value was used (p = 0.05) and interpretations were cautious. Only fingers that were identified as having a significant main effect of Session in the within-participants, two-way GEE analyses (see main text Results section, and Table 3 of the main text) were followed up with the time to learn analysis – as these were the only fingers for which there was a statistically verifiable change from in thresholds across sessions. Note these results are included here in the Supplementary Materials because we had no predictions regarding when particular fingers would show significant gains. This is a data-driven analysis, provided for interest only.

*Block only group*

The two-way GEE of the block only group (Finger x Session) revealed a significant main effect of the right middle finger only (see main text). Using the time to learn analysis, we found that the threshold of the right middle finger improved at the retention test (p = .006), though it was already trending towards improvement at the offline test (p = .096). This analysis suggests that the behavioural effects of temporary deafferentation may emerge and develop over the course of a few days. This suggestion is consistent with the findings of Shibata et al. (2012) who found deafferentation enhanced visual contrast sensitivity training between 5-8 days after the intervention, but not before or after this time. The delayed threshold drop we report here, however, may also simply indicate that more power is needed to detect a subtle change in perception, occurring earlier in the time-course. Please note, while this result indicates that gains are at their most different from baseline in the retention session for the deafferenation-adjacent finger, the difference between the deafferentation-adjacent finger and the remaining five fingers (averaged) is maximal at the offline session (see main text for details).

*Block+train group*

For the block+train group, the two-way GEE (Finger x Session) revealed five from six fingers tested showed a significant main effect of Session (see main text). These were the right index, right middle, right ring, left middle and left ring fingers (with no significant gains in the left index finger only). Once again, to probe the time-course of this learning, we performed the time to learn analysis for each of the fingers that showed a main effect. We found that the homologous left middle finger had already improved when tested during the online session (p = .004). The trained right middle finger (p = .002), right ring finger (p = .017), and left ring finger (p = .028) had significantly learned by the offline session. The deafferented right index finger (adjacent to the trained finger) only showed significant learning by the final retention session (p = .008). This apparent delay in learning may be due to deafferentation effects that vary over time (Merzenich et al., 1984) or to delayed transfer of tactile perceptual learning. Indeed, transfer in tactile learning has been shown not to always occur immediately, or necessarily at the same rate for all fingers. In our previous study (Dempsey-Jones et al., 2016), we show that while the trained finger learned on the day of training (i.e., showed significant change from baseline thresholds at this time), learning transfer did not occur until one or two days following the initial training (varying by finger). Thus, a delay in transfer is not unexpected. Particularly, in the deafferented right index finger, central or peripheral effects due to deafferentation might have interfered with normal processes of learning transfer to exaggerate normal lags in learning transfer.

*Sham+train group*

As there were no significant main effects of Session (i.e., no threshold values that differed significantly from each other, see main text), the time to learn analysis was not performed for the sham+train group.

**Part VIII. Discussion of the locus of the transfer of perceptual learning within the somatosensory system**

As discussed in the main text, the topographic organisation of the somatosensory system is integral to our interpretation of the pattern of transfer of perceptual learning. Some suggest perceptual learning occurs in sensory cortex e.g., through plastic changes in tuning properties of sensory neurons (Adab & Vogels, 2011; Jehee, Ling, Swisher, van Bergen, & Tong, 2012; Schoups, Vogels, Qian, & Orban, 2001; Shibata, Watanabe, Sasaki, & Kawato, 2011). These accounts predict tactile learning that either does not spread (Dinse et al., 2006, though see critique in Dempsey-Jones, 2016) or spreads in a topographic pattern (humans: Dempsey-Jones et al., 2016; Harrar et al., 2014; Harris et al., 2001; rodents: Harris & Diamond, 2000; Harris et al., 1999). Other theories suggest perceptual learning occurs in higher-order areas that *read-out* from sensory cortex e.g., parietal or decision making areas (Huang, Lu, & Dosher, 2012; Kahnt, Grueschow, Speck, & Haynes, 2011; Law & Gold, 2008; Petrov, Dosher, & Lu, 2005); see Harrar et al., (2014) for further discussion. Regardless of the precise locus of learning, transfer of tactile perceptual learning must occur as a function of topographic organisation (or reorganisation following deafferentation). Thus, the pattern of learning transfer we report here is consistent with several contemporary perceptual learning theories.

Transfer of tactile learning has also been suggested to occur ‘globally’ (to all fingers tested). However, many such studies only examine transfer to one finger, typically the finger homologous to (Kaas, van de Ven, Reithler, & Goebel, 2013; Nagarajan, Blake, Wright, Byl, & Merzenich, 1998; Sathian & Zangaladze, 1998; Spengler et al., 1997) or adjacent to the trained finger (Nagarajan et al., 1998; though see Arnold & Auvray, 2014). Such designs do not allow for separation between global and topographic accounts. Those that have attempted to dissociate these two drivers of transfer indicate the spread of spatial tactile learning is best characterised by SI topography (Dempsey-Jones et al., 2016; Harrar et al., 2014; Harris, Harris, & Diamond, 2001).
